# Supplementary material for: The feeder system of the Toba supervolcano from the slab to the shallow reservoir
Source: Nat Commun. 2016 Jul 19;7:12228. doi: 10.1038/ncomms12228 (PMC4960321; doi:10.1038/ncomms12228)
Supplement: Supplementary Information — Supplementary Figures 1-8 and Supplementary Tables 1-2 [file ncomms12228-s1.pdf]

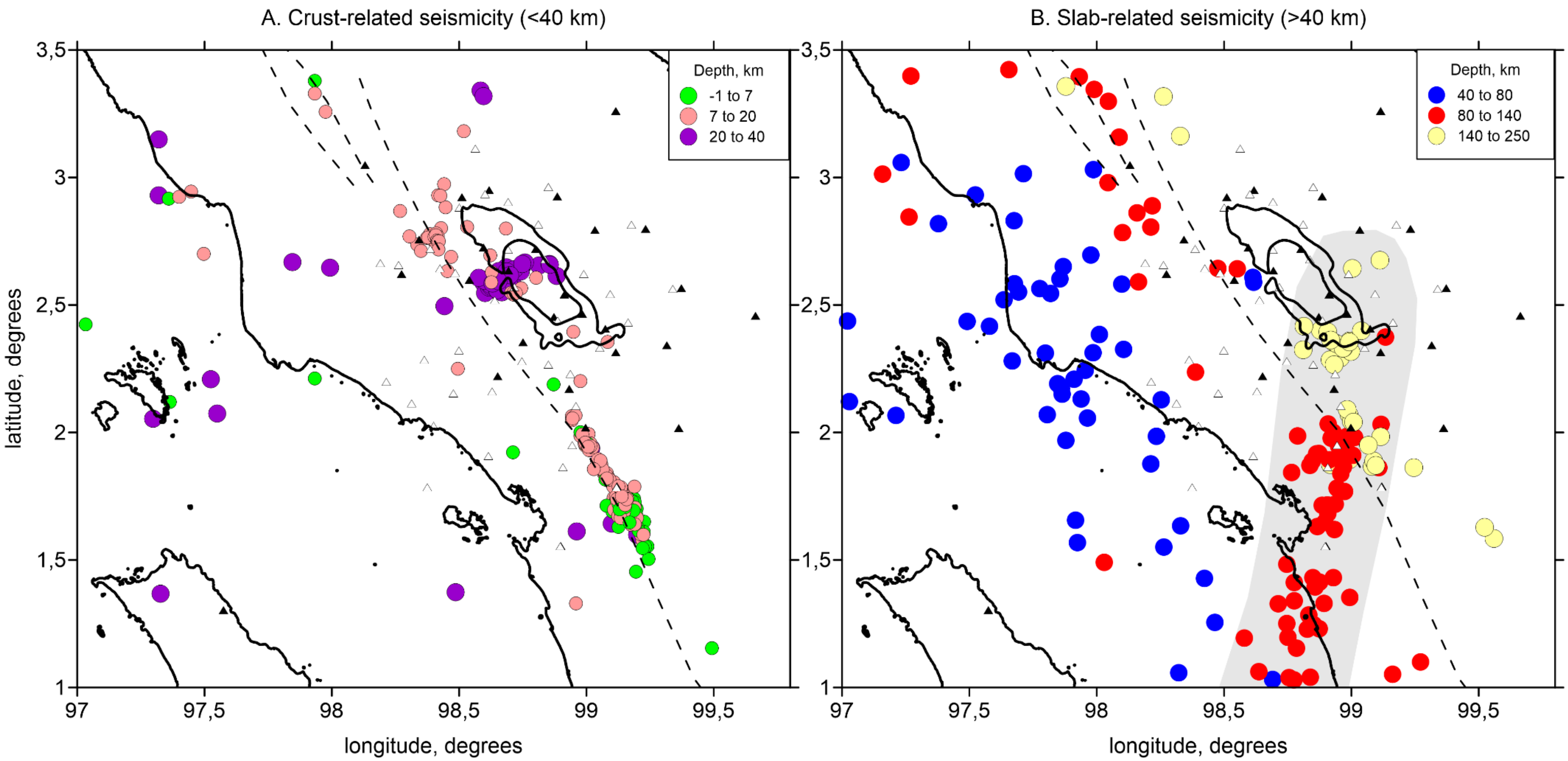

Supplementary Figure 1. Distribution of seismic event locations determined using the final 3-D velocity model. We separate the crust-related seismicity at depths of less than 40 km (panel A) from the deeper events associated with the slab (panel B). Colors of the events represent the depth ranges according to the scale. Black and white triangles depict seismic stations deployed in 1995 and 2008, respectively. The dotted line is the location of the Great Sumatran Fault Zone (GSFZ). The gray area highlights a zone of seismicity associated with the subduction of the Investigator Fracture Zone (IFZ).

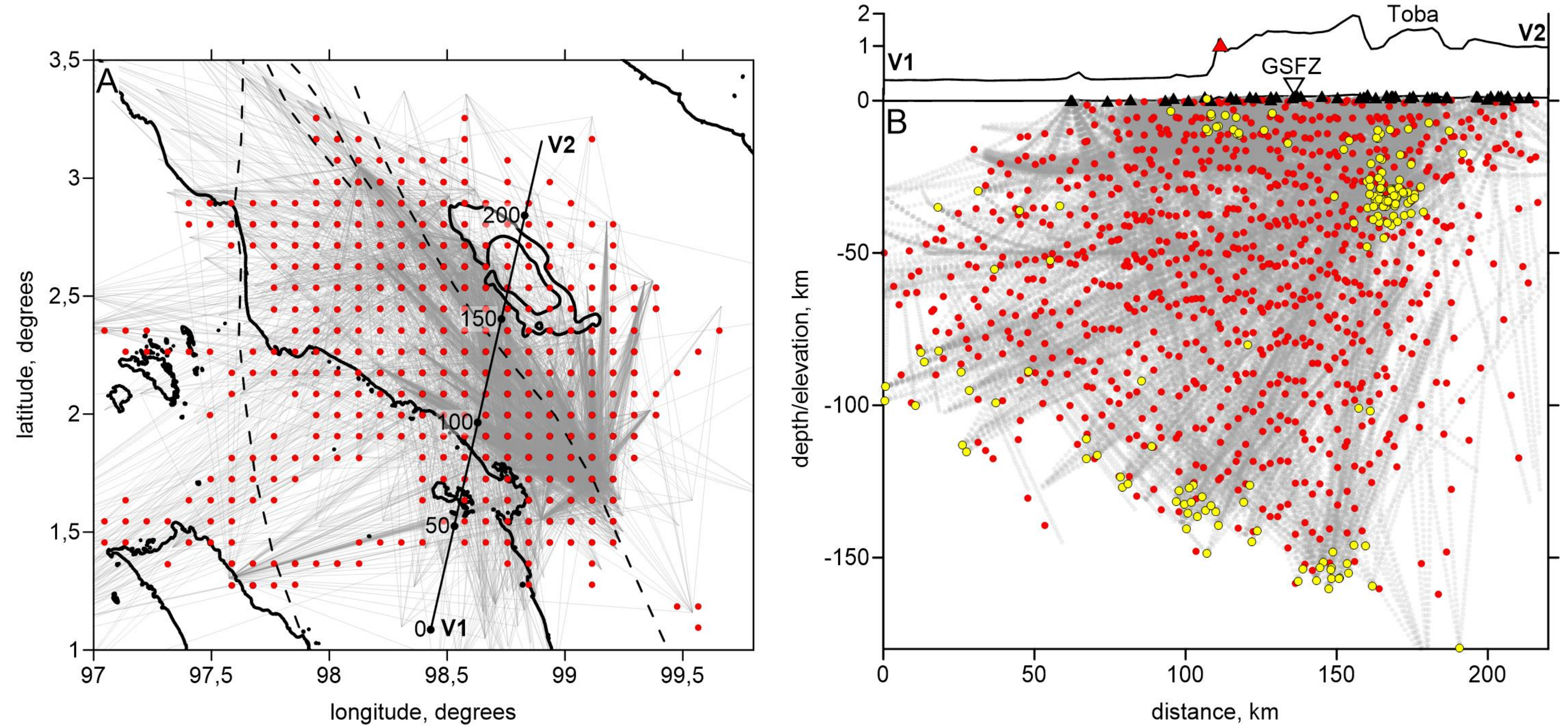

Supplementary Figure 2. Ray paths (gray lines/dots) and parameterization grid (red dots) in map view and a vertical section. The location of the profile is shown in the map view. Yellow dots depict events projected to the vertical section; black triangles are projections of seismic stations to the profile. The exaggerated relief along the profile is shown above the section.

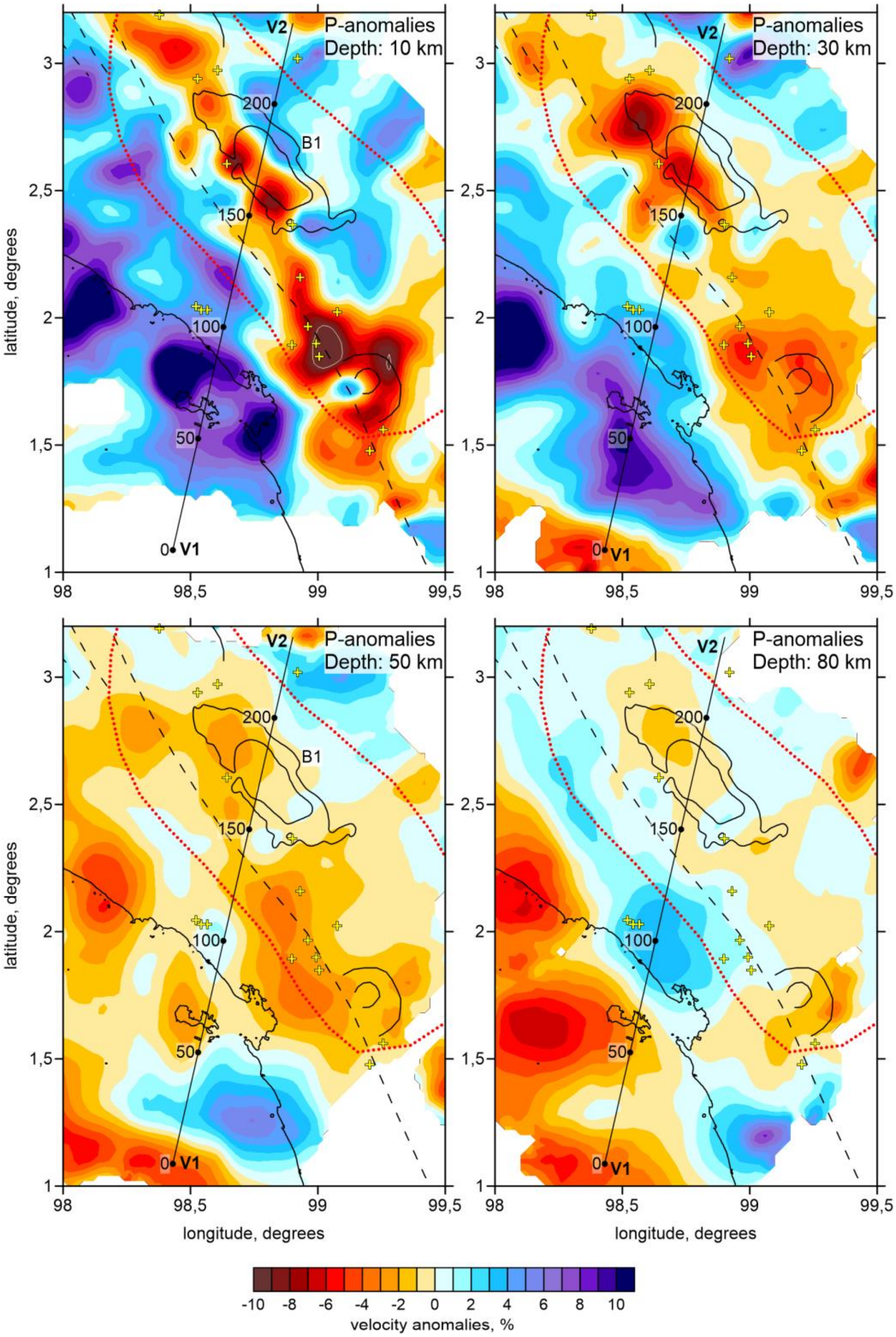

Supplementary Figure 3. P-wave velocity anomalies in four depth sections. The red dotted line indicates the uplift area. Crosses are the volcanic cones, and lines are caldera-related structures. The dashed line depicts the Great Sumatran Fault Zone (GSFZ). Profile V1–V2 indicates the location of the vertical section presented in Supplementary Figure 4.

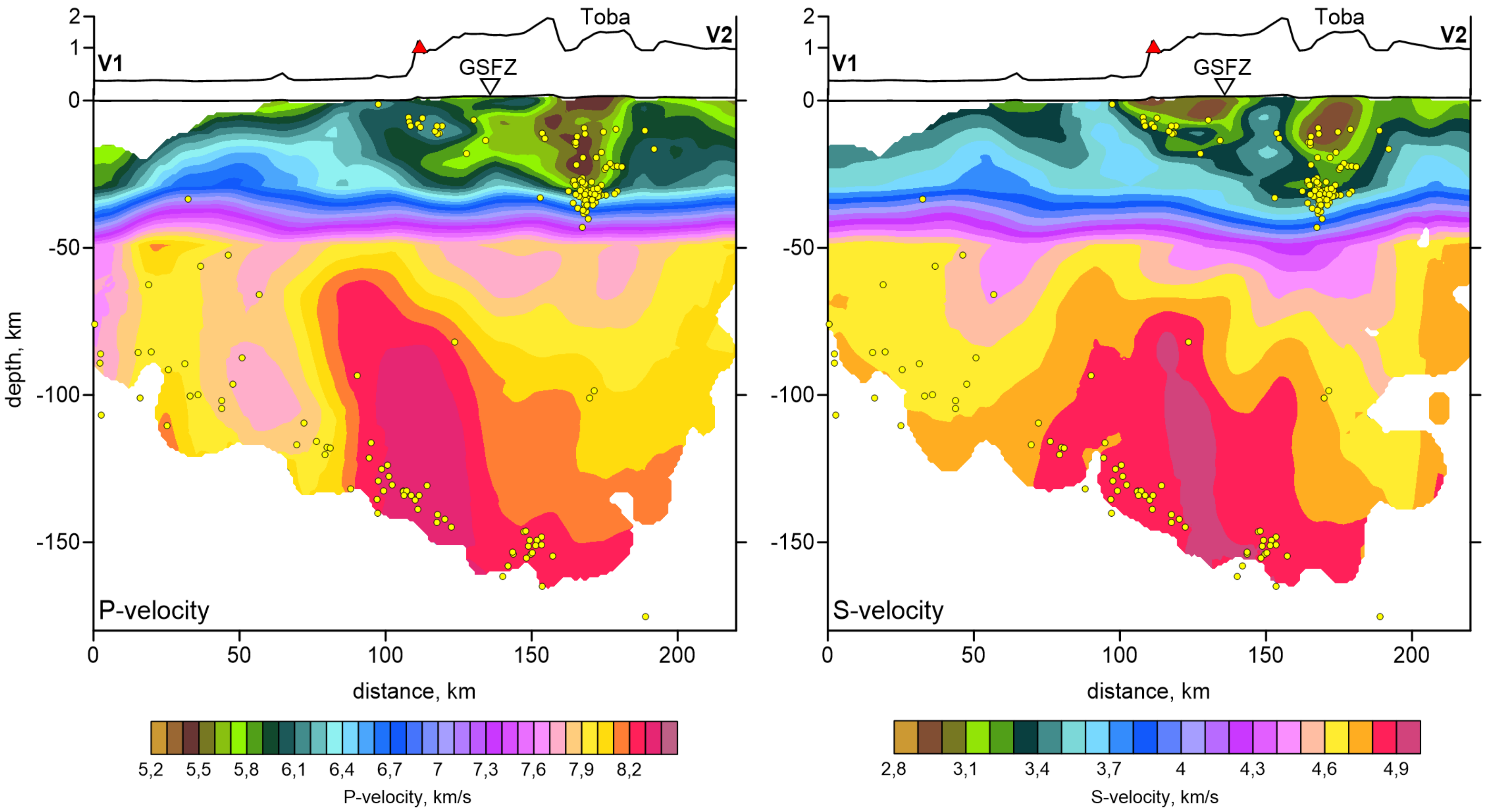

Supplementary Figure 4. Absolute P- and S-wave velocities in in the vertical section V1–V2 (location indicated in Supplementary Figure 3). Yellow dots depict seismic events located at distances of less than 20 km from the profile. The exaggerated relief is presented above each plot. The inverted triangle labeled GSFZ is the intersection with the Great Sumatran Fault Zone. The red triangle marks a volcanic complex identified in the coastal area.

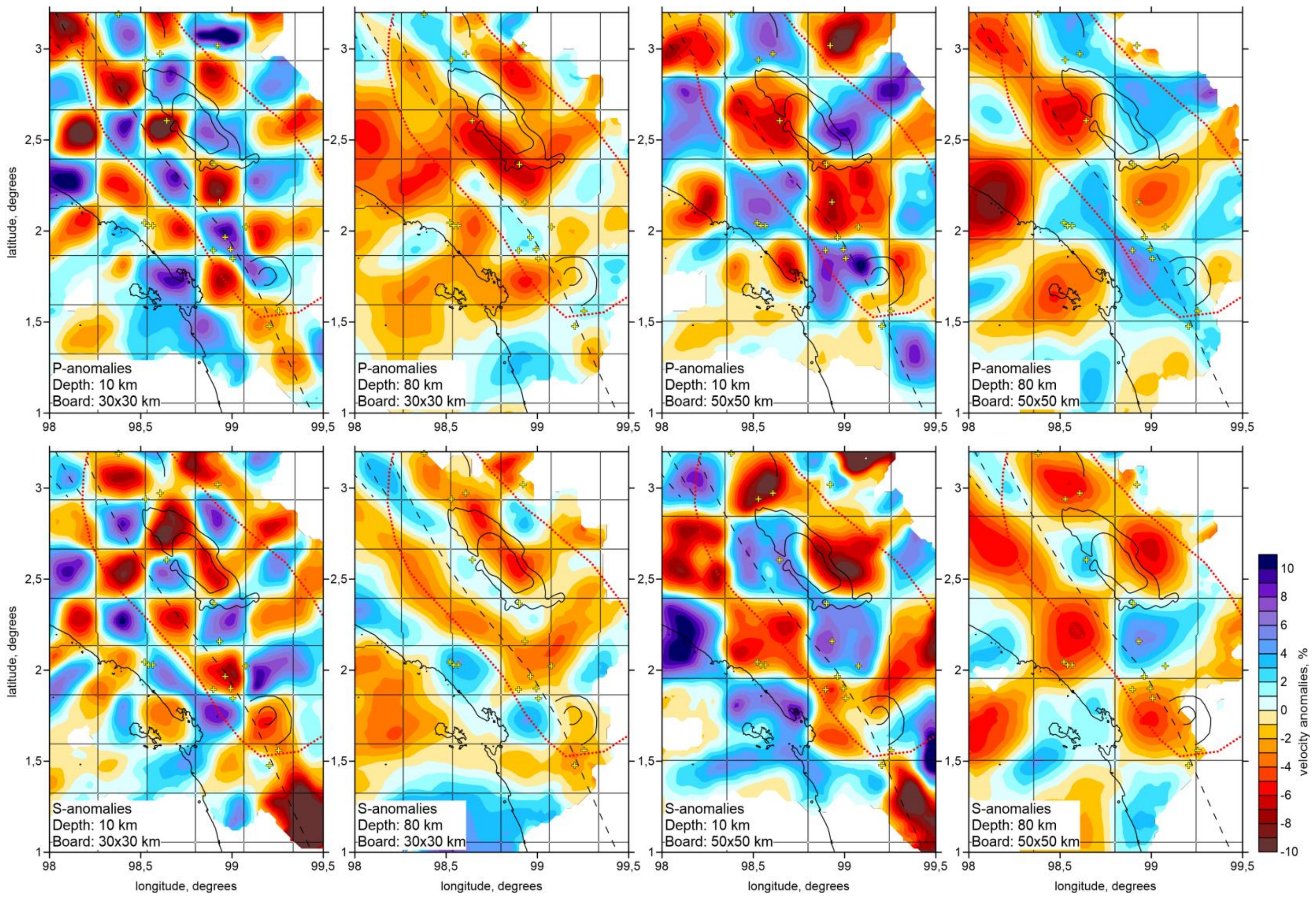

Supplementary Figure 5. Results of two checkerboard tests with the spacing of anomalies equal to 30 km and 50 km. The recovered P- and S-wave velocity anomalies are presented at 10 km and 80 km depth. Thin contour lines delineate the initial synthetic patterns. All of the structural elements are the same as in Figure 1B.

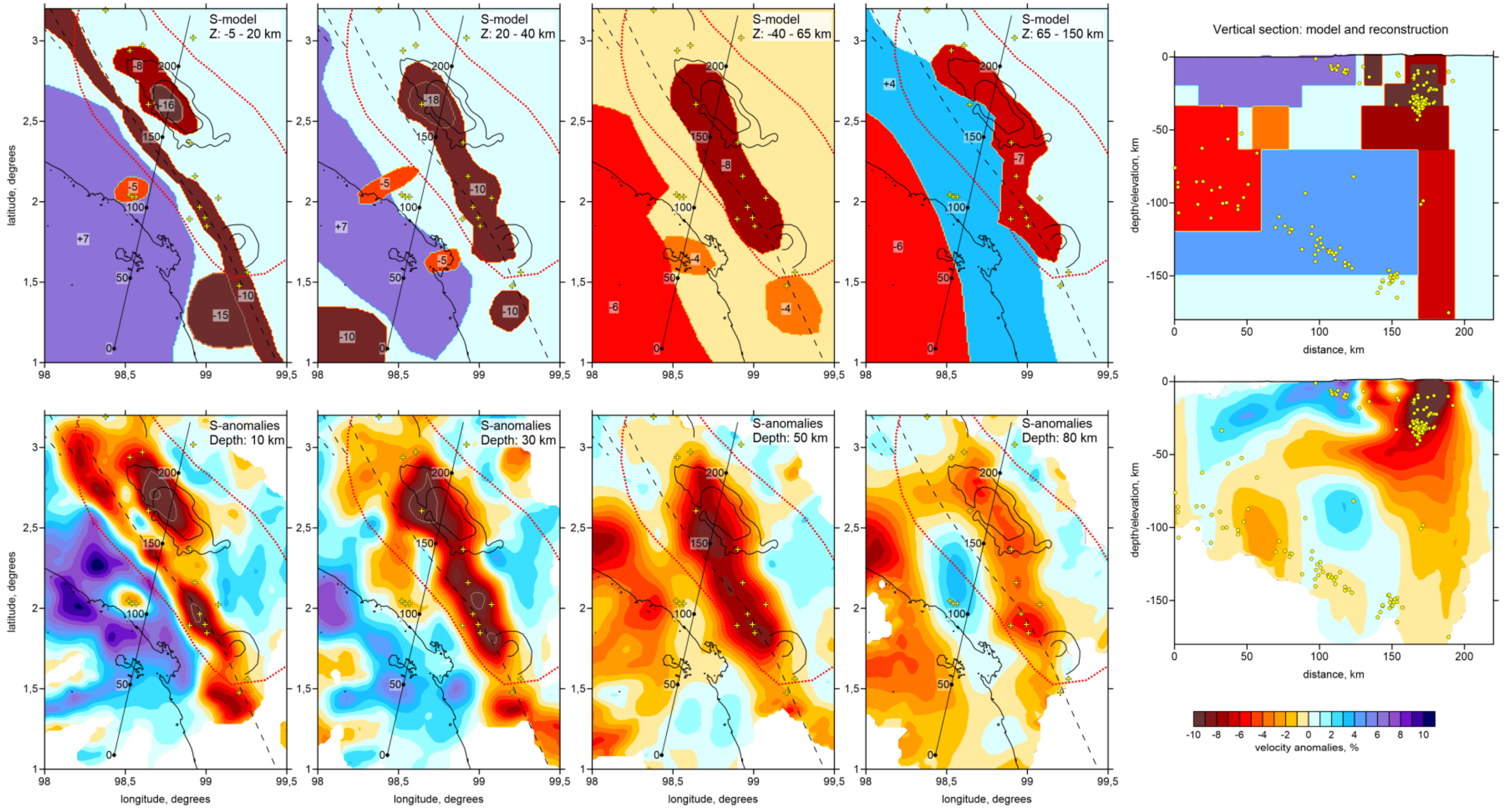

Supplementary Figure 6. Synthetic modeling results of the S-model. The configurations of synthetic anomalies at four depth intervals and in one vertical section are presented in the upper row. The recovery results for the S-wave anomalies at four depths and in the vertical section are presented in the lower row. All of the structural elements are the same as in Figure 1B.

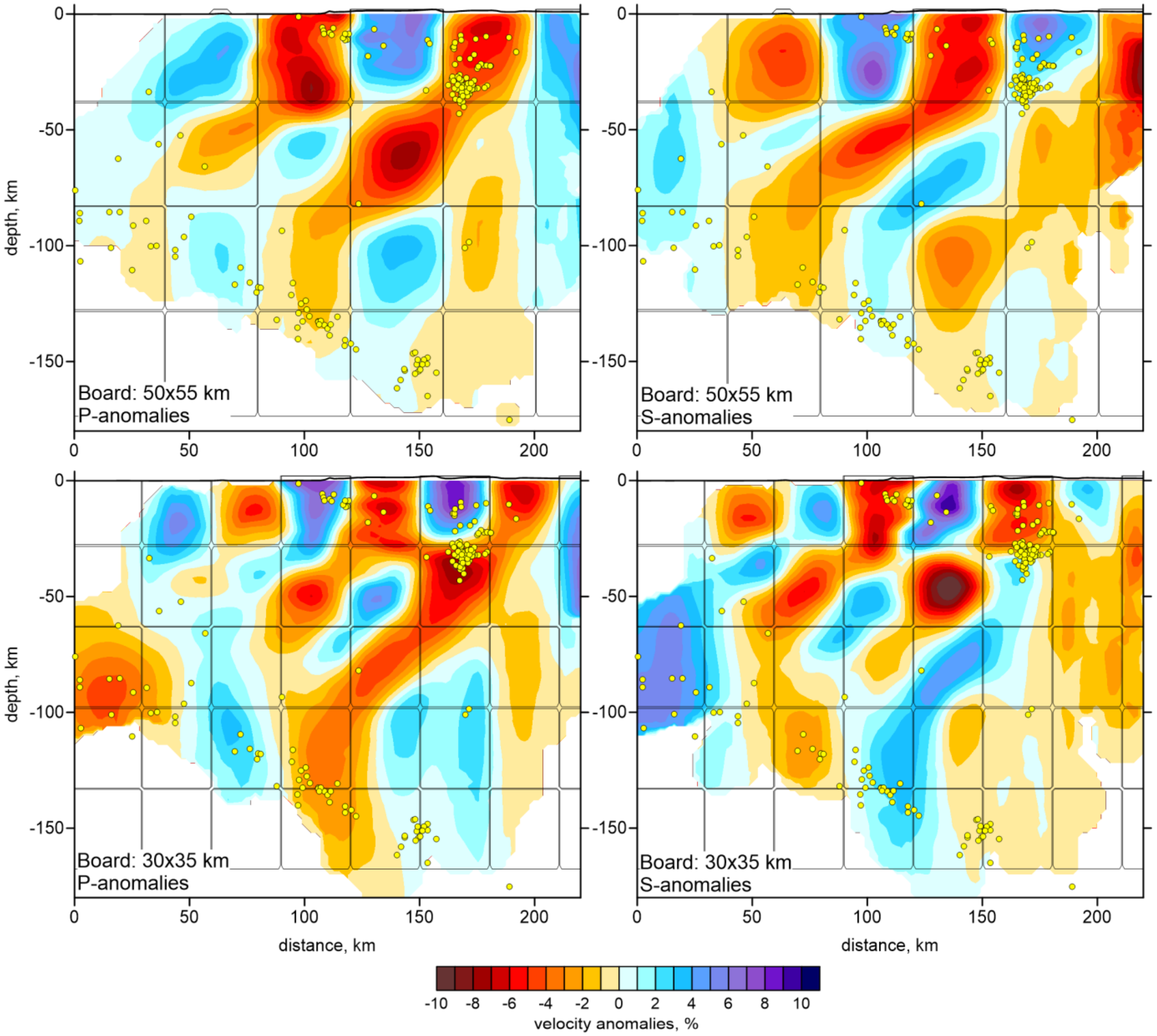

Supplementary Figure 7. P- and S-wave velocity anomalies of two checkerboard models with synthetic anomalies defined in a vertical section (same as that used for presenting the main results in Supplementary Figure 4). Thin black contour lines indicate the locations of the synthetic patterns. Yellow dots depict the locations of events.

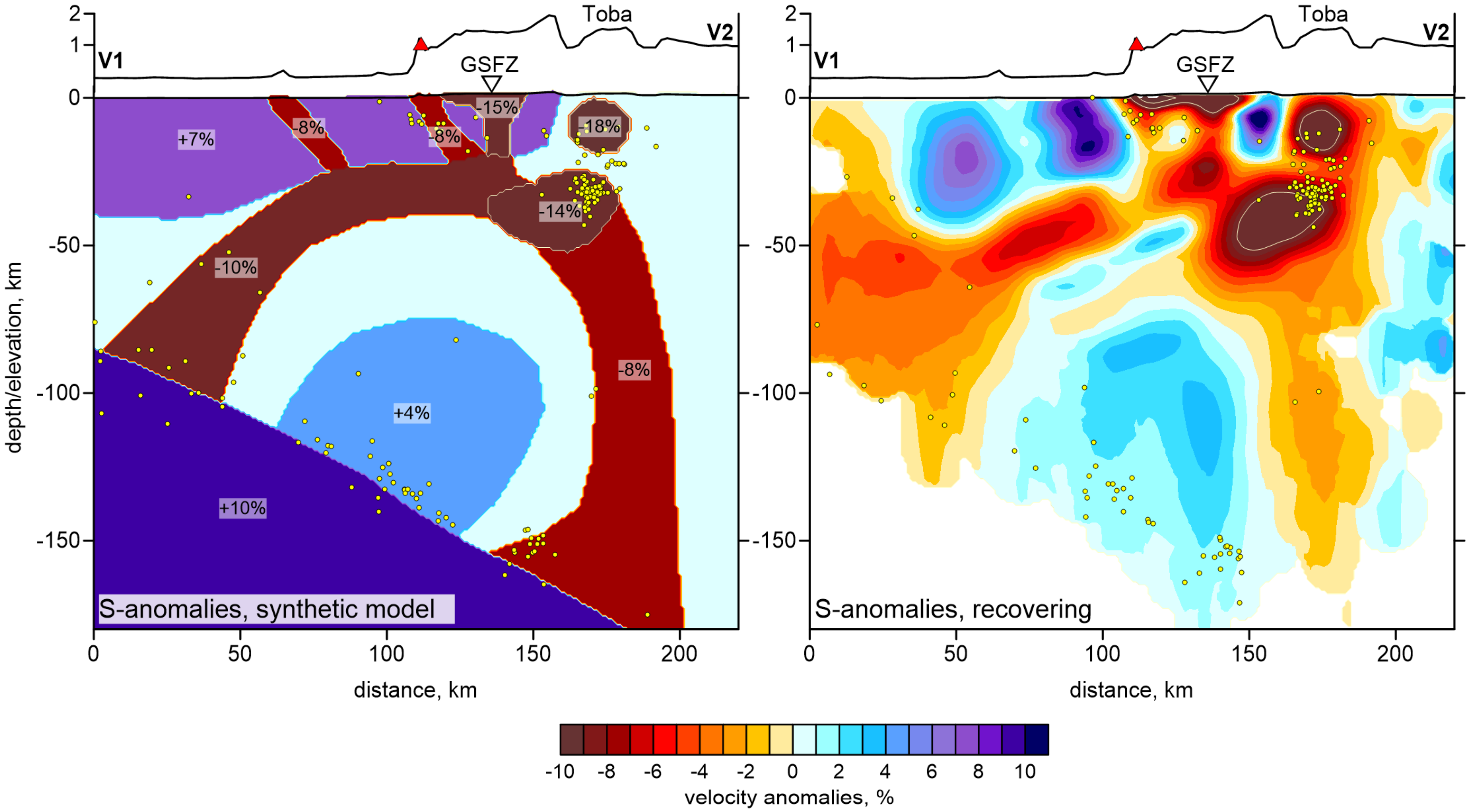

Supplementary Figure 8. Synthetic test with a realistic configuration of anomalies in the vertical section V1–V2 (same as that used for presenting the main results in Figure 4 of the main paper). The left plot represents the synthetic patterns with indications of the amplitudes of anomalies. The right plot shows the recovered result for the S-velocity structures. The exaggerated topography is shown above each plot. GSFZ is the Great Sumatran Fault Zone. Yellow dots mark the locations of events.

Supplementary Table 1. Reference one-dimensional velocity model[1] used for the tomographic inversion. S-velocity is derived using the constant Vp/Vs=1.7.

Depth, km	P-wave velocity, km/s
-1	5.5
30	6.1
50	7.9
120	8.1
1000	11.5

Supplementary Table 2. Values of average deviations of residuals and variance reduction during five iterations of tomographic inversion for the main model.

Iteration	Average residual deviation, s		Variance reduction, %	
	P-data	S-data	P-data	S-data
1	0.390	0.612	0.0	0.0
2	0.291	0.394	25.2	35.5
3	0.270	0.362	30.6	40.7
4	0.262	0.351	32.7	42.6
5	0.256	0.343	34.2	43.8

Reference:

[1]Koulakov, I., Yudistira, T., Luehr, B. G. & Wandono. P, S velocity and Vp/Vs ratio beneath the Toba caldera complex (northern Sumatra) from local earthquake tomography. *Geophys. J. Int.* **177**, 1121–1139, (2009).
